# 

**Published:** 1906-09-15

**Authors:** 


### BOOKS RECEIVED.

C. Griffin and Co.

"Treatment of Diseases of the Digestive System." By R. Saundby, M.D.

Hodder and Stoughton.

"Genito-Urinary and Venereal Disease." By L. E. Schmidt, M.D.

P. S. King and Co.

"Report of the Proceedings of the National Conference on Infantile Mortality."

Baillière, Tindall, and Cox.

"Clinical Studies in the Treatment of the Nutritional Disorders of Infancy." By R. Vincent, M.D.

Adam and Charles Black.

"Tuberculosis: Its Origin and Extinction." By W. Pickett Turner, M.D.

David Bryce and Son, Glasgow.

"A System of Surgical Nursing." By A. McGregor, M.D. M.D.

Cassell and Co., Ltd.

"The Other Side of the Lantern." By Sir Frederick Treves.

H. K. Lewis.

"A Guide to the Administration of Ethyl Chloride." By G. A. H. Barton, M.D.

J. B. Lippincott Co.

"Clinical Diagnosis." By C. P. Emerson, M.D.

Sherratt and Hughes, Manchester.

"Practical Prescribing and Dispensing." By William Kirkby.

### NOTICE TO CORRESPONDENTS.

**All MSS., Letters, Books for Review, and other matters intended for the Editor should be addressed THE EDITOR, "THE HOSPITAL," THE HOSPITAL BUILDINGS, 28 & 29 SOUTHAMPTON STREET, STRAND, W.C.**

**The Editor cannot undertake to return rejected MS., even when accompanied by stamped directed envelope.**

**Notice to Advertisers and Subscribers.**

**All Advertisements, Orders for Copies of THE HOSPITAL, and Business Communications should be addressed to THE MANAGER (Not to the Editor), THE HOSPITAL, 28 & 29 Southampton Street, Strand London, W.C.**

**The Cost of Subscription, post free, is as follows:—Per week, 3½d.; per quarter, 3s. 3d.; per half-year, 6s. 6d.; per annum, 13s. Foreign and Colonial:—Per annum, 19s. 6d., payable, in all cases, in advance.**

### ART OF FEEDING THE INVALID.

By a Medical Practitioner and a Lady Professor of Cookery.

New and Popular Edition, with Special Chapter on Feeding in Consumption. 1/6 post free.

### DIET IN SICKNESS AND IN HEALTH.

By Mrs. Ernest Hart. With an Introduction by Sir Henry Thompson, F.R.C.S., M.B., Lond. 3/6 post free.

### ELEMENTARY TREATISE ON THE LIGHT TREATMENT FOR NURSES.

By James H. Sequeira, M.D. Lond. Illustrated. 2/6 net, 2/9 post free.

### HINTS TO NURSES ON TROPICAL FEVERS.

By S. F. Pollard, Sister Army Nursing Reserve, late Sister Guy's Hospital.

The Work is based upon the Personal Experience of the Author Abroad. 1/6 net, 1/8 post free.

### INSTRUCTIONS TO MIDWIVES.

Compiled by the Midwives Supervising Committee of the Corporation of Manchester. 6d. net, 7d. post free.

### LECTURES ON HOME NURSING FOR THE POOR.

By a District Nurse. Illustrated. 1/6 net, 1/8 post free.

### LECTURES UPON THE NURSING OF INFECTIOUS DISEASES.

By F. J. Woollacott, M.A., M.D. 2/6 net, 2/9 post free.

### MEDICAL ELECTRICITY AND THE LIGHT TREATMENT.

By Kate Neale, Sister in Charge Light Department, Guy's Hospital. Illustrated. 2/6 net, 2/9 post free.

### NURSING IN DISEASES OF THE THROAT, NOSE AND EAR.

By P. Macleod Yearsley, F.R.C.S. Eng., M.R.C.S., L.R.C.P. Lond. Illustrated. 2/6 post free.

### OPHTHALMIC NURSING.

By Sydney Stephenson, M.B., F.R.C.S.E.

New and Revised Edition profusely Illustrated, and a Glossary of Terms. 3/6 net, 3/10 post free.

### OUTLINES OF INSANITY.

**A Popular Treatise on the Salient Features of Insanity.**

By Francis H. Walmsley, M.D. Popular Edition, stitched. 1/6 post free.

### SIMPLE INTRODUCTORY LESSONS ON MIDWIFERY.

By M. Loane. Illustrated. 1/- net, 1/1 post free.

### SIMPLE SANITATION:

**The Practical Application of the Laws of Health to Small Dwellings.**

By M. Loane. With Introductory Chapter on Sanitary Legislation by D. A. Mearns Proser. 1/- net, 1/2 post free.

### THE CARE OF CHILDREN.

**Practical Hints for Mothers and Nurses at Home and Abroad.**

By Robt. J. Blackham, D.P.H. Lond., C.B. Revised and Enlarged Edition. 1/6 net, 1/8 post free.

### THE HUMAN BODY.

**Its Personal Hygiene and Practical Physiology.**

By B. P. Colton, Professor of Natural Science, Illinois University. Profusely Illustrated. 5/- post free.

### THE MODERN NURSING OF CONSUMPTION.

By Dr. Jane H. Walker. 1/- net, 1/2 post free.

☞ ***Catalogue of Nursing Manuals sent post free on application.*** ☜

**The Scientific Press, Ltd.,**

**Publishers of Nursing Text-Books, Charts, and Case-Books of all Descriptions.**

**28 & 29 SOUTHAMPTON STREET, STRAND, LONDON, W.C.**

### Publications.

JUST PUBLISHED. Price **2s. 6d.** net.

**CARCINOMA OF THE RECTUM.**

By F. SWINFORD EDWARDS, F.R.C.S.,

Surgeon to the West London Hospital; Senior Surgeon to St. Peter's Hospital for Stone, and to St. Mark's Hospital for Fistula, &c.

London: Baillière, Tindall, & Cox, 8 Henrietta Street, Covent Garden.

THE MOST EFFECTUAL BANDAGE FOR THE CURE OF ULCERS AND OTHER DISEASES OF THE LEG.

**NOTES ON GENERAL PRACTICE.**

By S. M. HEBBLETHWAITE, M.D. Lond.

"Sound common sense."—*Hospital.* "We can heartily recommend."—*B. M. Journal.* "A shrewd practical knowledge of everyday general practice."—*Lancet.* "Well worth perusal."—*Scottish Med. & Surg. Journal.* "A great deal of sound advice."—*Edinburgh Med. Journal.* "Will not fail to pleasantly interest."—*Glasgow Med. Journal.* "Well fulfils his hope that they may be found useful."—*Medical Review.* "A very great deal indeed of excellent clinical information."—*Dublin Journal of Med. Sciences.*

Post free 3/9. THE SCIENTIFIC PRESS 28 Southampton St., W.C.

**WORKS BY DAVID WALSH, M.D.**

**RÖNTGEN RAYS IN MEDICAL WORK.** *Third Edition.* 12s. 6d.
**THE HAIR AND ITS DISEASES.** 2s. 6d. net. A Standard Monograph. Baillère, Tindall, & Cox, London.
**GOLDEN RULES OF SKIN PRACTICE.** 1s. net. *Second Edition.* Wright & Co., Bristol.
**AGE AND OLD AGE: Handbook.** 2s. 6d. R. A. Everett & Co., London.

**MEDICAL HISTORY FROM THE EARLIEST TIMES.**

By E. T. WITHINGTON, M.A., M.B.Oxon.

Demy 8vo., over 400 pp., with two Plates, cloth gilt, 12/6 net.

"One of the best attempts that has yet been made in the English language to present the reader with a concise epitome of the history of medicine, and as such we very cordially commend it."

*Glasgow Medical Journal.*

THE SCIENTIFIC PRESS, Ltd.,

28 and 29 Southampton Street, Strand, London, W.C.

